# Black liver in a patient with Wilson's disease

**DOI:** 10.1002/ccr3.6513

**Published:** 2022-11-06

**Authors:** Wei Jiang, Qingmin Zeng, Chang‐Hai Liu, Dongbo Wu, Hong Tang

**Affiliations:** ^1^ Center of Infectious Diseases West China Hospital of Sichuan University Chengdu China

**Keywords:** copper metabolism, iron overload, liver biopsy, Wilson disease

## Abstract

Wilson's disease is an autosomal recessive inherited disease with congenital copper metabolism disorder, characterized by decreased ceruloplasmin and increased urine copper, which can involve multiple organs. This case was complicated by iron overload, which is of great value in differentiating hereditary hemochromatism.

## Abstract 2

When Wilson's disease presents increased serum iron and liver iron deposition, liver MRI shows black liver image, and liver pathology shows iron deposition in zone 2–3. At this time, it is necessary to identify with hereditary hemochromatism.

A 37‐year‐old man with elevated alanine aminotransferase (ALT) for 6 years was repeatedly misdiagnosed. The laboratory investigations showed ALT 171.9 IU/L, ceruloplasmin 0.023 g/L, ferritin 1218.8 ng/ml, and normal urine copper. The head MRI scan and Kayser–Fleischer rings were negative, while abdominal MRI scan showed T2‐weighted imaging with loss of signal intensity in the liver (Figure 1A). Liver biopsy suggested steatohepatitis with macrovesicular and microvesicular steatosis (Figure 1B), and iron overload in zone 2–3 hepatocytes and Kupffer cells, with Perls' staining grade of 2–3 (Figure 1C). The hepatic copper and iron concentrations were 274.3 and 1769.2 μg respectively. Genetic detection reports presented the Glu1173Lys mutations with c.3517G>A in the exon16 of ATP7B gene, without mutations in hemochromatosis gene, so he was diagnosed with Wilson disease (WD) and secondary iron overload.

**FIGURE 1:**
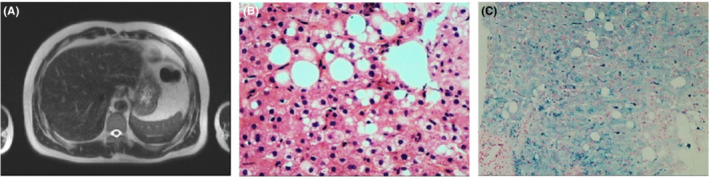
(A) Abdominal MRI scans revealed the loss of signal intensity in the liver in T2‐weighted imaging; (B) Histologic examination of liver biopsy suggested steatohepatitis with macrovesicular and microvesicular steatosis on hematoxylin–eosin staining (×10); (C) Perls' Prussian blue staining was grade of 2–3 with iron overload in zone 2–3 hepatocytes and Kupffer cells (×10).

Wilson disease is characterized by copper metabolism disorder caused by the mutations of ATP7B gene, manifested as decreased ceruloplasmin and increased urine copper, which can simultaneously or solely involve the liver, eyes, and nervous system.^1^ The character of increased ferritin and iron overload in liver in WD patients is rarely reported, which may have been due to the lack of ferroxidase activity, and it is liable to be mistaken for hereditary hemochromatosis.^2^

## AUTHOR CONTRIBUTIONS

Wei Jiang, Qingmin Zeng, and Chang‐Hai Liu collected the data and drafted the manuscript. Dongbo Wu and Hong Tang made critical revisions to the manuscript for important intellectual content. All authors approved the final version of the manuscript.

## FUNDING INFORMATION

It was supported by the Science and Technological Supports Project of Sichuan Province, China (No. 2019YSF0028 and 2021YFS0169), Post‐Doctor Research Project, West China Hospital, Sichuan University (grant number 2018HXBH005), and National Natural Science Foundation of China (No. 81900512).

## CONFLICT OF INTEREST

None declared conflict of interest.

## ETHICAL APPROVAL

Personal data have been respected.

## CONSENT

Written informed consent was obtained from the patients to publish.

## Data Availability

Data available on request due to privacy/ethical restrictions.
